# Supplementary material for: The ubiquitin-proteasome system regulates the formation of specialized ribosomes during high salt stress in yeast
Source: bioRxiv. 2024 Aug 15:2024.08.15.608112. Preprint. [Version 1] doi: 10.1101/2024.08.15.608112 (PMC11343215; doi:10.1101/2024.08.15.608112)
Supplement: Supplement 1 [file NIHPP2024.08.15.608112v1-supplement-1.pdf]

bioRxiv preprint doi: https://doi.org/10.1101/2024.08.15.608112; this version posted August 15, 2024. The copyright holder for this preprint (which was not certified by peer review) is the author/funder, who has granted bioRxiv a license to display the preprint in perpetuity. It is made available under aCC-BY 4.0 International license.

Table S1: Yeast strains used in this work

Strain	Description	Background	Genotype	Reference
YKK200	WT	BY4741	*MATα his3Δ1 leu2Δ0 met15Δ0 ura3Δ0*	GE Dharmacon
YKK491	Gal::Rps26	BY4741	*MATα NatMX6::pGAL1-Rps26A Rps26B::KanMX6 his3Δ1 leu2Δ0 met15Δ0 ura3Δ0*	(Ferretti et al 2017)
YKK1650	ΔGID4	BY4741	*MATα gid4:: GID4::KanMX6 his3Δ1 leu2Δ0 met15Δ0 ura3Δ0*	GE Dharmacon
YKK1651	ΔGID10	BY4741	*MATα gid4:: GID10::KanMX6 his3Δ1 leu2Δ0 met15Δ0 ura3Δ0*	GE Dharmacon

Table S2: Plasmids used in this work

Plasmid	Description	Backbone	Reference
pKK3558	TEF::Rps26A	pRS416	(Ferretti et al 2017)
pKK31160	TEF::Rps26A_P2S	pRS416	This work
pkk30528	Gal::Rps26A-HA	pRS426	(Yang & Karbstein 2022)
pKK31150	Gal::Rps26A_P2S-HA	pRS426	This work
pKK31155	Gal::Rps26A_D33N-HA	pRS426	This work
pKK31157	Gal::Rps26A_P2SD33N-HA	pRS426	This work
pKK31177	Gal::Rps26A_K28R-HA	pRS426	This work
pKK31178	Gal::Rps26A_K66R-HA	pRS426	This work
pKK31179	Gal::Rps26A_K70R-HA	pRS426	This work
pKK31180	Gal::Rps26A_K108R-HA	pRS426	This work
pKK31181	Gal::Rps26A_K116R-HA	pRS426	This work
pKK31042	Gal::Rps3-HA	pRS425	This work
pKK31195	TEF::Rps26A_K66R	pRS416	This work
pKK31196	TEF::Rps26A_K70R	pRS416	This work
